# Cloning, Expression and Characterization of Mitochondrial Manganese Superoxide Dismutase from the Whitefly, *Bemisia tabaci*

**DOI:** 10.3390/ijms14010871

**Published:** 2013-01-07

**Authors:** Xian-Long Gao, Jun-Min Li, Yong-Liang Wang, Min Jiu, Gen-Hong Yan, Shu-Sheng Liu, Xiao-Wei Wang

**Affiliations:** 1 Ministry of Agriculture Key Laboratory of Agricultural Entomology, Institute of Insect Sciences, Zhejiang University, Hangzhou 310058, China; E-Mails: gxl060308024@163.com (X.-L.G.); wangyongliang2006a@126.com (Y.-L.W.); ghyan@zju.edu.cn (G.-H.Y.); shshliu@zju.edu.cn (S.-S.L.); 2 Institute of Virology and Biotechnology, Zhejiang Academy of Agricultural Sciences, Hangzhou 310021, China; E-Mail: zjlijunmin@gmail.com; 3 College of Food and Bioengineering, Henan University of Science and Technology, Luoyang 471023, China; E-Mail: jiumin0912@163.com

**Keywords:** mitochondrial manganese superoxide dismutase, oxidative stress, whitefly, *Bemisia tabaci*

## Abstract

A mitochondrial manganese superoxide dismutase from an invasive species of the whitefly *Bemisia tabaci* complex (Bt-mMnSOD) was cloned and analyzed. The full length cDNA of Bt-mMnSOD is 1210 bp with a 675 bp open reading frame, corresponding to 224 amino acids, which include 25 residues of the mitochondrial targeting sequence. Compared with various vertebrate and invertebrate animals, the MnSOD signature (DVWEHAYY) and four conserved amino acids for manganese binding (H54, H102, D186 and H190) were observed in Bt-mMnSOD. Recombinant Bt-mMnSOD was overexpressed in *Escherichia coli*, and the enzymatic activity of purified mMnSOD was assayed under various temperatures. Quantitative real-time PCR analysis with whiteflies of different development stages showed that the mRNA levels of Bt-mMnSOD were significantly higher in the 4th instar than in other stages. In addition, the *in vivo* activities of MnSOD in the whitefly were measured under various conditions, including exposure to low (4 °C) and high (40 °C) temperatures, transfer from a favorable to an unfavorable host plant (from cotton to tobacco) and treatment with pesticides. Our results indicate that the whitefly MnSOD plays an important role in cellular stress responses and anti-oxidative processes and that it might contribute to the successful worldwide distribution of the invasive whitefly.

## 1. Introduction

The whitefly *Bemisia tabaci* (Gennadius) (Hemiptera: Aleyrodidae) is a species complex with global distribution, and some members of the complex cause severe damage to the production of vegetables, fibers and ornamental crops [1–4]. A major recent event associated with *B. tabaci* has been the global invasion of Middle East-Asia Minor 1 specie (MEAM1, formerly known as the “B biotype”) that has been ranked among the top 100 invasive species [5–7]. MEAM1, one of the 24 putative species delineated by Dinsdale *et al.* [8], invaded China in the mid-1990s [9–11] and gradually displaced indigenous *B. tabaci* species during its invasion in many regions [7,12]. Previous studies indicated that the invasive ability of MEAM1 may be closely related to its high capability of surviving under different stress conditions [1,13]. And recently, reports showed that the activity of superoxide dismutase might be associated with the ability of invasive whitefly to survive under various stress conditions, such as extreme temperatures and unfavorable host plants [14,15].

Superoxide dismutases (SODs) are a family of metalloenzymes that catalyze the dismutation of reactive oxygen species (ROS) [16], which can be toxic to nucleic acids, proteins and membrane lipids [17,18]. Three major types of SODs have been characterized based on their metal content: copper/zinc SOD (CuZnSOD), manganese SOD (MnSOD) and iron SOD (FeSOD) [19,20]. They are widely distributed in bacteria, fungi, plants and animals [21].

MnSOD is primarily found in prokaryotes and eukaryotic mitochondria [22]. Two types of MnSOD are known in eukaryotes: mitochondrial MnSOD (mMnSOD), with a mitochondrial transit peptide for translocation, and cytosolic MnSOD (cMnSOD) without the peptide [23]. In eukaryotic cells, mMnSOD is synthesized as a precursor protein in the cytosol and then imported into the mitochondrial matrix [21]. The mMnSOD is located in mitochondria and plays a vital role in defense against superoxide radicals generated as byproducts of oxidative phosphorylation [24]. cMnSOD has been identified and cloned in various crustacean species. The characterization and its role in immunomodulation have also been reported [19,25,26]. Preliminary studies showed that the overexpression of MnSOD can extend the life span of adult *Drosophila melanogaster* [27] and reduce oxidative stress in cells [28,29]. Recently, it was found that MnSOD may be involved in immune responses to stimulators, such as heat, coldness, starvation and heavy metals [30,31]. However, until now, information about the function of MnSOD in *B. tabaci* was still lacking.

Previously, we found that CuZnSOD plays a major role in protecting the whitefly *B. tabaci* against various stress conditions [15]. To gain further insights into the characteristics of MnSOD and its functional roles in the defense system, we cloned the mMnSOD gene of *B. tabaci* and purified the recombinant protein expressed in *Escherichia coli*. We then performed biochemical assays to elucidate the activity of the recombinant protein. Next, we examined the mRNA levels of Bt-mMnSOD in different developmental stages of the whitefly. Finally, we investigated the *in vivo* activities of MnSOD in the whitefly under various stress conditions including heat, coldness, shift of host plants and pesticide treatments.

## 2. Results

### 2.1. Analysis of the Bt-mMnSOD Sequence and the Predicted Protein

**Figure 1:**
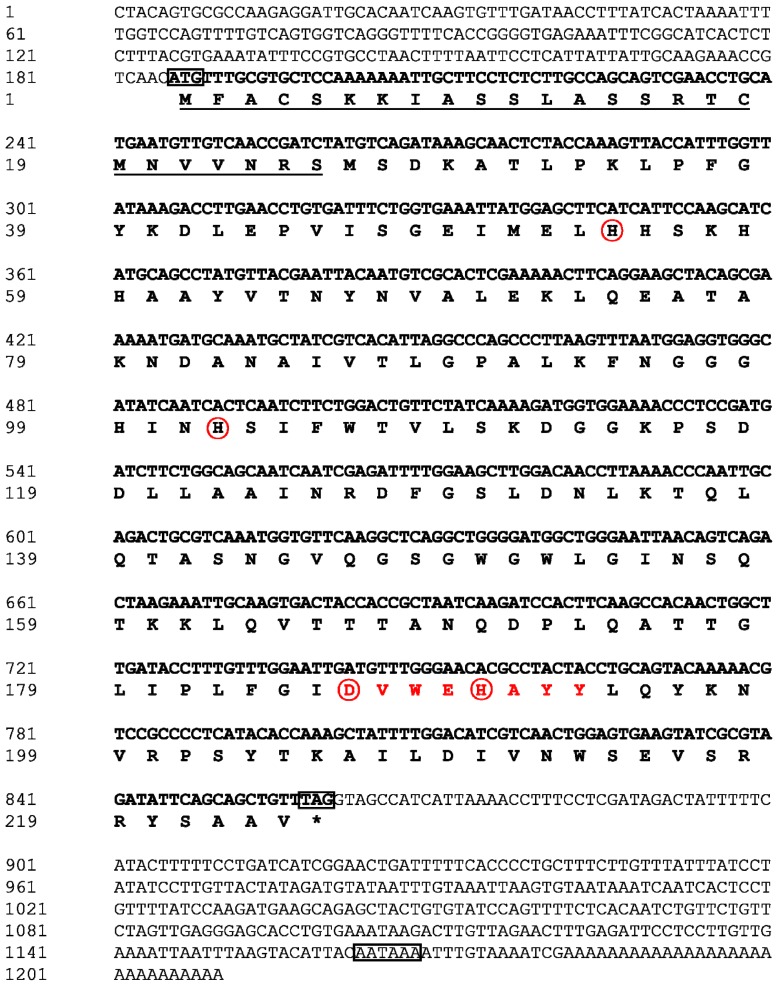
Nucleotide and deduced amino acid sequences of mitochondrial manganese superoxide dismutase from *Bemisia tabaci* (Bt-mMnSOD). The start codon (ATG), stop codon (TAG) and putative polyadenylation signal (AATAAA) are boxed. Putative mitochondrial targeting sequence is underlined. The manganese superoxide dismutase (MnSOD) signature motif (DVWEHAYY) is shown in bold red letters. Four putative manganese binding sites (H54, H102, D186 and H190) are circled.

The full-length mMnSOD cDNA of *B. tabaci* (GenBank accession number JQ867105) consists of 1210 bp, with a 675 bp open reading frame (ORF), which encodes 224 amino acids. The full-length nucleotide sequences and the deduced amino acid sequences are shown in Figure 1. The Bt-mMnSOD cDNA sequence contains a 5′ untranslated region (UTR) of 185 nucleotides, a long 3′ UTR of 350 nucleotides consisting of a stop codon (TAG), a putative polyadenylation consensus signal (AATAAA) and a poly (A) tail. The predicted molecular mass is 24.5 kDa and the estimated isoelectric point of this protein is 8.97. SignalP program analysis showed no putative signal peptide in Bt-mMnSOD. However, TargetP and MITOPROT revealed that it contains a putative mitochondrial targeting sequence of 25 amino acids and is located inside the mitochondria.

**Figure 2:**
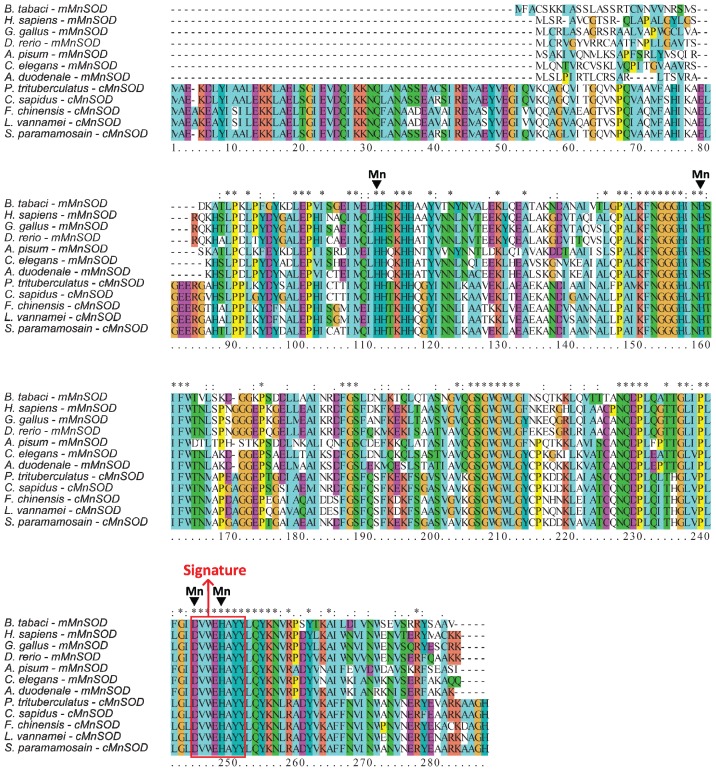
Multiple alignments of the deduced amino acid sequence of the Bt-mMnSOD with the mitochondrial MnSOD (mMnSODs) and cytosolic MnSOD (cMnSODs) of other species. The manganese superoxide dismutase signature DVWEHAYY is boxed in red (labeled Signature), and the amino acids required for binding Mn are indicated with arrows.

Multiple sequences alignment of Bt-mMnSOD with other MnSODs of vertebrate and invertebrate animals indicated high conservation of four putative manganese binding sites (H54, H102, D186 and H190) and a MnSOD signature from 186 to 193 (DVWEHAYY) (Figure 2). The major differences between the mitochondrial and cytosolic MnSODs (cMnSODs) are located at the *N*-terminal region (Figure 2). cMnSODs have a conserved *N*-terminal extension, which is not present in mMnSODs, but lack a mitochondrial targeting sequence. MnSOD sequences of whitefly and other species were obtained from GenBank (Table 1).

**Figure 3:**
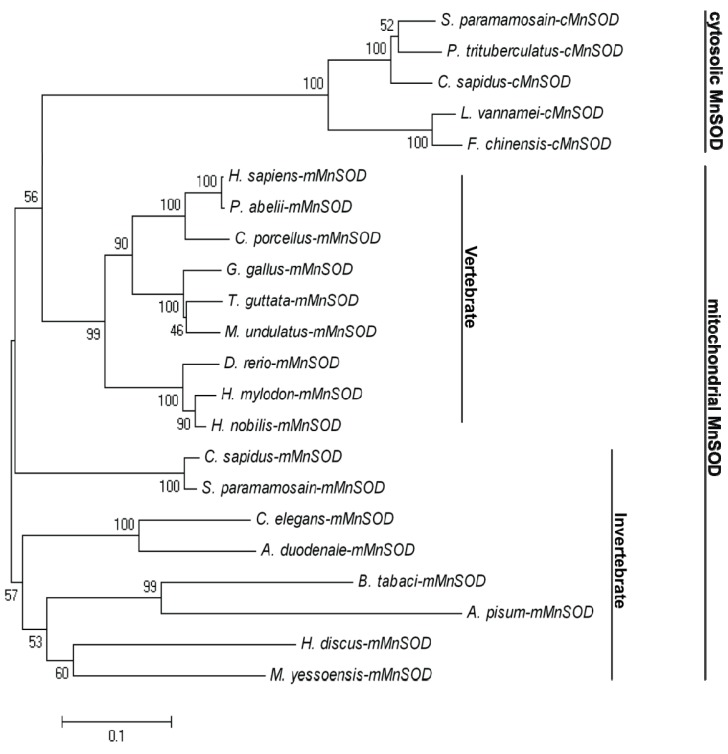
Phylogenetic relationships between mMnSODs and cMnSODs from 22 different species. A phylogenetic analysis was conducted based on a multiple alignment of amino acid sequences retrieved from the GenBank database. Numbers at the nodes are bootstrap values (1000 replications). The scale bar is 0.1.

A phylogenetic tree of the amino acid sequences of selected MnSODs was constructed by the neighbor-joining distance method using MEGA version 4 (Figure 3). The phylogenetic analysis indicated that MnSODs can be classified into two groups. All the mMnSODs were clustered together as one subgroup and all cMnSODs as another group (Figure 3). Bt-mMnSOD was in the same group of other mMnSODs, suggesting that it is an mMnSOD.

### 2.2. Overexpression and Purification of Bt-mMnSOD

**Figure 4:**
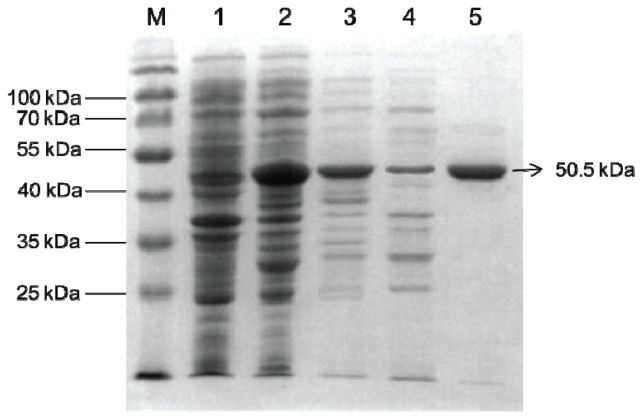
Expression and purification of superoxide dismutase (SOD) recombinant protein. Lane M, molecular weight marker; lane 1, un-induced total protein; lane 2, total protein induced with 0.2 mM isopropyl β-d-1-thiogalactopyranoside (IPTG) for 18 h at 18 °C; lane 3, insoluble proteins; lane 4, soluble proteins; lane 5, purified recombinant protein of GST-Bt-mMnSOD.

The expression of Bt-mMnSOD protein was considerably increased after induction with 0.2 mM IPTG at 18 °C for 18 h (Figure 4, lanes 1, 2). Moreover, the proteins were expressed both in the inclusion bodies and as soluble protein (Figure 4, lanes 3, 4). The soluble Bt-mMnSOD protein was then purified with GST purification system and resolved by SDS-PAGE, with the result of a 50.5 kDa single band (Figure 4, lane 5). The molecular mass of the purified product was consistent with the predicted molecular weight of the recombinant protein, which consisted of Bt-mMnSOD (24.5 kDa) and GST tag (26 kDa).

### 2.3. Thermostability of Purified Bt-mMnSOD

**Figure 5:**
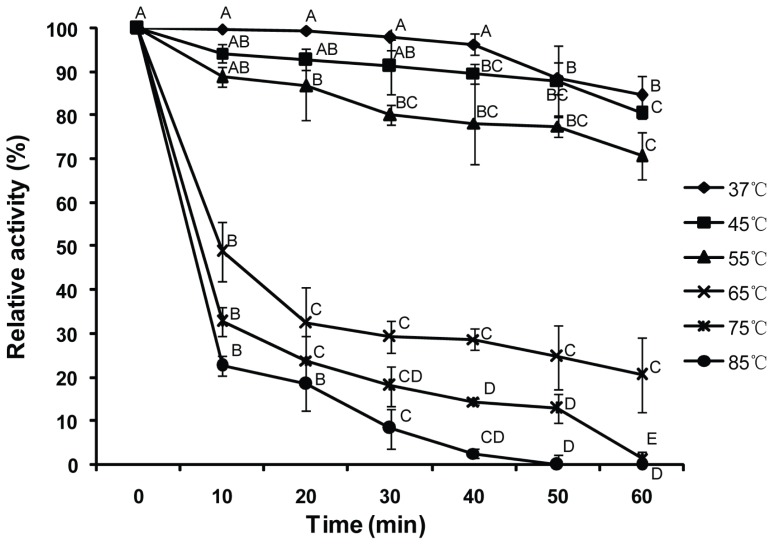
Effects of temperature on the purified recombinant Bt-mMnSOD activity. Each bar represents the mean ± SD (*n* = 3). For each temperature, significant differences are indicated by different letters at *p* < 0.01 (least significant difference (LSD) test).

Thermostability of purified Bt-mMnSOD was studied by incubating the enzyme at 37, 45, 55, 65, 75 and 85 °C for various intervals. The residual activities of Bt-mMnSOD were measured (Figure 5). The enzymatic activity of Bt-mMnSOD was stable after incubation at 37 °C and 45 °C for 30 min. At the temperature of 55 °C, the Bt-mMnSOD activity decreased significantly after 20 min (*p* < 0.01), but still retained up to 70% after 60 min incubation. The activity reduced by 50% when incubated for 10 min at 65 °C and decreased rapidly in the initial 10 min (*p* < 0.01) at 75 and 85 °C. The enzyme was completely inactivated when incubated for 50 min at 85 °C and 60 min at 75 °C, respectively.

### 2.4. Expression of Bt-mMnSOD during Development

**Figure 6:**
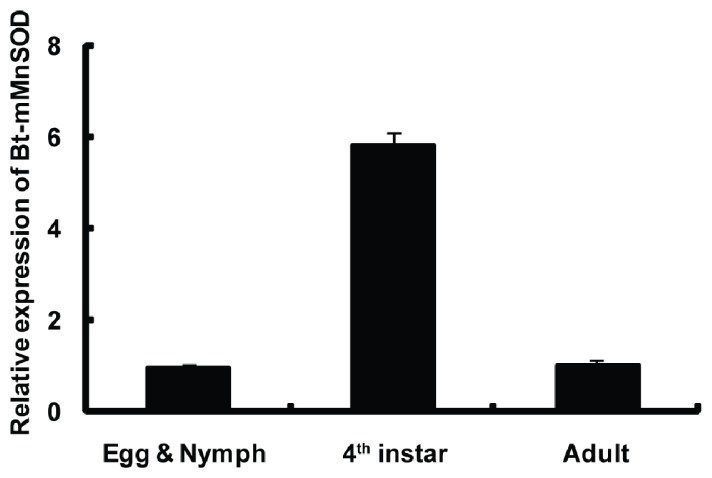
Quantification of mitochondrial manganese zinc superoxide dismutase from *Bemisia* (Bt-mMnZnSOD) gene expression in different developmental stages. Each symbol and vertical bar represent the mean ± SD (*n* = 3).

To better understand the physiological roles of Bt-mMnSOD, its expression was detected by qPCR in different developmental stages. The mRNA levels of Bt-mMnSOD were significantly higher in the 4th instar than in egg and nymph or adult (Figure 6). The result of qPCR suggests that Bt-mMnSOD might play important roles in the development of *B. tabaci*, especially in the 4th instar.

### 2.5. Effect of Thermal Stresses, Host Shift and Pesticide Treatment on MnSOD Activity

**Figure 7:**
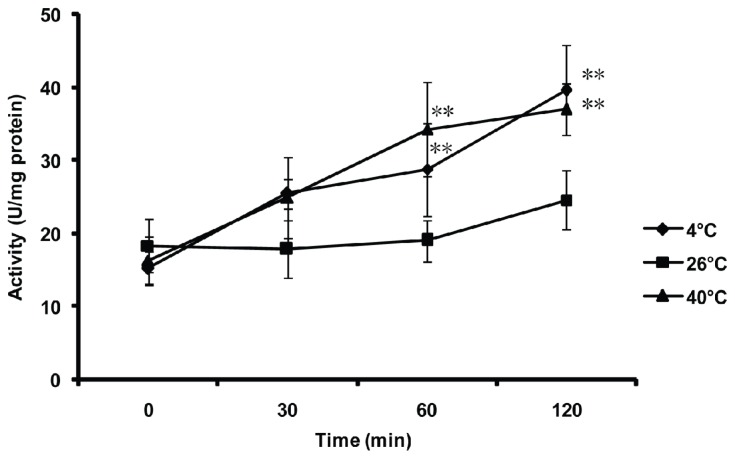
Effect of thermal stresses to MnSOD activity of whitefly *in vivo*. Each bar represents the mean ± SD of MnSOD activity (*n* = 3). Compared with control (0 min), significant differences of each temperature at different sampling times are indicated by two asterisks at *p* < 0.01 (LSD test).

To evaluate the *in vivo* activity of the MnSOD in *B. tabaci* stimulated by external temperature stresses, *B. tabaci* adults were exposed to low (4 °C), medium (26 °C) and high (40 °C) temperatures for 0 (control), 30, 60 and 120 min, respectively (Figure 7). The activity of MnSOD significantly increased after incubation at low (4 °C) and high (40 °C) temperatures for 60 and 120 min, compared with control (*p* < 0.01). However, no significant differences were detected when the *B. tabaci* adults were exposed at 26 °C from 0 to 120 min or incubated at 4 °C and 40 °C in the initial 30 min.

**Figure 8:**
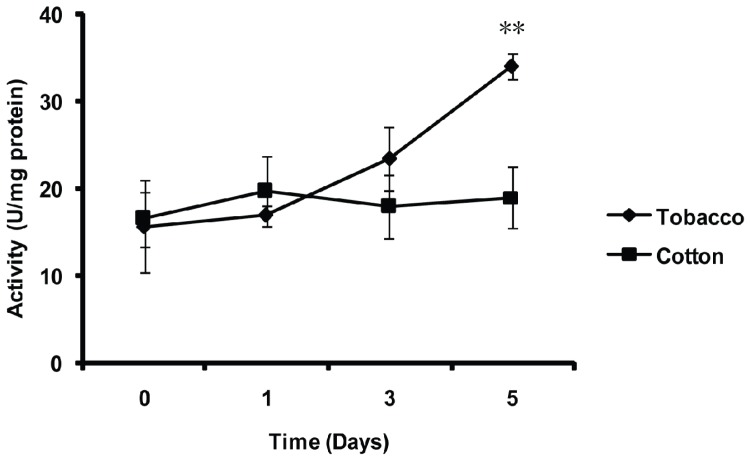
Effect of host shift on MnSOD activity of whitefly *in vivo*. Each symbol and bar represents the mean ± SD of MnSOD activity (*n* = 3). Significant differences between various sampling times of each host plant compared with 0 days are indicated by two asterisks at *p* < 0.01 (LSD test).

To characterize the effect of host shift on the *in vivo* activity of the MnSOD in *B. tabaci*, whitefly adults were transferred from cotton (a favorable host plant) to tobacco (an unfavorable host plant) for 0, 1, 3 and 5 days. When whiteflies were transferred from cotton to tobacco for 5 days, the activity of MnSOD significantly increased compared with 0 days (*p* < 0.01), whereas in the control (transfer from cotton to cotton), the level of activity remained unchanged (Figure 8).

**Figure 9:**
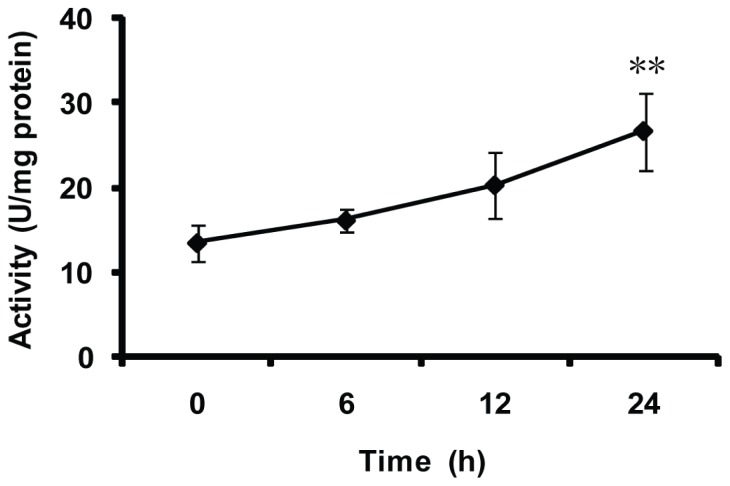
Effect of pesticide on MnSOD activity of whitefly *in vivo*. Each bar represents the mean ± SD (*n* = 3). Significant differences between various times compared with 0 h are indicated by two asterisks at *p* < 0.01 (LSD test).

Furthermore, the activities of MnSOD were measured after the whitefly feeding on the imidacloprid-treated cotton plants for various durations (Figure 9). No significant differences were observed in the initial 12 h. However, after 24 h feeding on imidacloprid-treated cotton plants, a significant increase of MnSOD activity was detected compared with 0 h (*p* < 0.01).

## 3. Discussion

Reactive oxygen species are formed in the body as natural products of oxidative metabolism. ROS cause oxidative stress and can be toxic to many cell components [17]. The mitochondrial matrix is the major site for the production of superoxide radicals. MnSOD is known to be a key antioxidant enzyme in the mitochondrial matrix, which can catalyze the dismutation of the superoxide radicals into hydrogen peroxide [32,33].

MnSOD genes have been cloned from various organisms, but few data are available regarding the mMnSOD from *B. tabaci*. In the present study, a mMnSOD cDNA was identified from *B. tabaci.* Multiple sequences alignment of mMnSODs and cMnSODs from various species shows that the four metal-binding residues and MnSOD signature are highly conserved, indicating that these sites are essential to the structure and function of MnSODs. The identification of the signature sequence and conserved metal-binding residues suggested that the Bt-mMnSOD possesses the essential properties of MnSOD family. MITOPROT analysis revealed it contains a putative mitochondrial targeting sequence of 25 amino acids, which can translocate the mMnSOD into the mitochondrial matrix [21,23]. For phylogeny analysis, MnSODs from different species were found to cluster into two groups with robust separate branches of cMnSOD and mMnSOD. Bt-mMnSOD clustered with the mMnSOD group, suggesting that it is a mitochondrial MnSOD.

Temperature stress was reported as one of the key mediators of ROS generation [34,35]. Previous studies have shown that temperature stress can cause MnSOD induction in several species: Chinese hamster lung fibroblast cell [36], *Hydra vulgaris* [37] and *Hyphantria cunea* [30]. An earlier study indicated that the activity of CuZnSOD in *B. tabaci* was noticeably enhanced under cold and heat stress [15]. In this study, we found that the MnSOD activity of *B. tabaci* was also increased with the aforementioned conditions. These results indicated that both MnSOD and CuZnSOD may function synergistically as scavengers to remove the intracellular superoxide anions generated under high or low temperatures, which might be related to the MEAM1 invasive ability.

Species of phytophagous insects differ in preference, ingestion speed, digestion efficiency and detoxification of the ingredients ingested towards different host plants, leading to the variability in their ability of host utilization [38,39]. Therefore, host shift might affect the individual performance of insects. In our study, we found that the *in vivo* activity of MnSOD was significantly increased compared with 0 days (*p* < 0.01) after transfer from a favorable plant (cotton) to an unfavorable plant (tobacco). Our result is consistent with previous studies of *Lymantria dispar* SOD [40] and *B. tabaci* CuZnSOD [15]. We speculate from this result that MnSOD may assist the whitefly MEAM1 to adapt to new host plants, which might contribute to its global invasion.

Insecticides can induce oxidative stress, which is always a big challenge for insects. Zang *et al.* [41] compared the susceptibility of MEAM1 and indigenous ZHJ1 population of whitefly to imidacloprid and pyriproxyfen and found that ZHJ1 was more susceptible to insecticides than MEAM1. The low susceptibility to insecticides of MEAM1 may contribute to the whitefly’s invasion and displacement of indigenous competitors. Interestingly, in our study, significant increase of MnSOD activity was found when MEAM1 fed on the cotton treated with imidacloprid. Similarly, Kemal [42] reported that low concentrations of malathion (0.01–1 ppm) resulted in the significantly increased activity of SOD in *Pimpla turionellae* females compared with control. Altogether, we speculate that the MnSOD of whitefly MEAM1 might be involved in response to pesticides. In addition, the displacement of indigenous whitefly species by MEAM1 might be related to MnSOD activities, which can reduce the high level of superoxide radically induced by pesticides.

## 4. Experimental Section

### 4.1. Whitefly Cultures and Plants

Stock cultures of the invasive whitefly MEAM1, collected from Zhejiang, China, were established in the laboratory. Details of the methods for maintaining whitefly cultures were described by Jiu *et al.* [43]. The cultures were maintained on cotton plants (*Gossypium hirsutum* L. cv. Zhemian 1793) and regularly monitored for purity using random amplified polymorphic DNA polymerase chain reaction (RAPD-PCR) with the primer H16 (5′-TCTCAGCTGG-3′) [44].

Cotton (cv. Zhemian 1793) and tobacco (*Nicotiana tabacum* cv. NC89), a suitable and an unsuitable host plant for the whitefly, respectively [45,46], were used in this study. All plants were cultivated with a potting mix (a mixture of peat moss, vermiculite organic fertilizer and perlite in a 10:10:10:1 ratio by volume) in plastic pots in whitefly-proof glasshouses at 27 ± 1 °C, 14 h light: 10 h darkness and 40%–60% relative humidity (RH). Plants were used for experiments at the 5–6 true leaf stage.

### 4.2. cDNA Synthesis and Cloning of the Bt-mMnSOD

Total RNA was isolated using the SV total RNA isolation system (Promega, Madison, WI, USA), following the manufacturer’s protocol. cDNA was synthesized using total RNA as a template with the SMART RACE cDNA Amplification Kit (Clontech, Palo Alto, CA, USA). Initially, partial Bt-mMnSOD cDNA sequence was obtained from the whitefly transcriptome deposited in NCBI (GenBank accession no. HP662057) [47]. The 5′ and 3′ ends of Bt-mMnSOD were amplified using the SMART RACE cDNA Amplification Kit with the 5′ primer (5′-CTAAACAGCTGCTGAATATC-3′) and 3′ primer (5′-ATGTTTGCGTGCTCCAAAAA-3′), following the manufacturer’s instructions. The amplified products were analyzed on 1.5% agarose gel, and the target band was purified by a PCR purification kit (Promega, Madison, WI, USA). Then the products were cloned into the pMD18-T vector (Takara, Dalian, China) for sequencing.

### 4.3. Sequence Analysis

The nucleotide and deduced amino acid sequences of the Bt-mMnSOD were analyzed by DNAMAN version 6 [48]. The sequence similarity of MnSODs from different species was analyzed and compared using the BLAST search program [49]. Theoretical isoelectric points and predicted molecular masses were calculated using Prot-Param tools [50]. The signal peptide was predicted by Signal P4.0 program [51]. The MITOPROT [52] and TargetP 1.1 [53] were used to predict whether the sequence contains any targeting sequence and where it locates. To compare MnSOD of whitefly and other species, related sequences were obtained from GenBank (Table 1) and aligned using Clustal W [54]. The phylogenetic relationship among the sequences was determined by reconstructing a protein phylogeny using MEGA4 [55]. The tree topology was evaluated by the bootstrapping method (1000 replications).

### 4.4. Overexpression and Purification of Bt-mMnSOD

A pair of primers were designed to clone the open reading frame of the Bt-mMnSOD and then ligated to the pGEX-4T-3 expression vector. The sense primer was designed as 5′-GGATCCATGTTTGCGTGCTCCAAAAA-3′ with a *Bam*H1 site and the antisense primer 5′-GTCGACCTAAACAGCTGCTGAATATC-3′ containing *Sal*1 site. The ligated product was then transformed into *E. coli* BL21 (DE3) pLysS cells for recombinant protein expression (Novagen, Madison, WI, USA).

Bacteria were grown in 800 mL LB medium containing 0.1 g/mL ampicillin at 37 °C until an optical density of 0.6 at 600 nm was reached. IPTG was added to a final concentration of 0.2 mM, and the culture was grown at 18 °C for 18 h for the induction of protein expression. Protein samples were analyzed by 12% SDS-PAGE, followed by staining with Coomassie brilliant blue. The Bt-mMnSOD protein with the GST tag was purified using the GST gene fusion system, as described [56]. The eluted protein content was measured by UV absorbance at 280 nm, and the purity of the protein was evaluated by 12% SDS-PAGE.

### 4.5. Thermostability of Purified Bt-mMnSOD

The activity of the purified recombinant Bt-mMnSOD was determined using the CuZn/Mn SOD Assay Kit (Beyotime, Shanghai, China). The kit applied the 2-(4-iodophenyl)-3-(4-nitrophenyl)-5-(2, 4-disulfophenyl)-2*H*-tetrazolium, monosodium salt (WST-1) method. The WST-1 could react with superoxide anion to generate water-soluble formazan dye. However, this reaction can be inhibited by SOD. Through colorimetric analysis of the WST-1 product, we can calculate the SOD enzyme activity. The absorbance was measured at 450 nm using spectrophotometer at room temperature. One unit of enzyme activity was defined as the amount of enzyme that results in 50% inhibition of the xanthine oxidase coupled reaction under the assay condition. To determine the thermal stability of Bt-mMnSOD, reactions were carried out at 37, 45, 55, 65, 75 and 85 °C for 0 (control), 10, 20, 30, 40, 50 and 60 min and the relative activity of Bt-mMnSOD was measured, respectively. Each treatment was replicated for three times. A multiple comparisons (LSD) test was conducted to detect significant differences (*p* = 0.01) between the treatments using the DPS software [57].

### 4.6. Bt-mMnSOD Gene Expression in Different Developmental Stages

Quantitative real-time PCR (qPCR) was performed on the ABI PRISM 7500 Fast Real-Time PCR System (Applied Biosystems, USA). Total RNA was isolated from different developmental stages of *B. tabaci*, including egg & nymph, 4^th^ instar and adult. RNA concentration was measured by NanoDrop 2000c (Thermo, Minneapolis, MN, USA). cDNA was synthesized using PrimeScript^®^ RT reagent Kit (Takara, Dalian, China). The following primers, qPCR-F (5′-ACCACCGCTAATCAAGATCC-3′) and qPCR-R (5′-TGCAGGTAGTAGGCGTGTTC-3′), designed by the GenScript Real-time PCR Primer Design Software [58], were used for qPCR. As an endogenous control, the expression of β-actin was measured in parallel.

### 4.7. Effect of Thermal Stresses on the MnSOD Activity of the Whitefly

Whiteflies were independently exposed to three different temperatures (4, 26, 40 °C) for 0, 30, 60 and 120 min in climatic chambers. The control and treated whiteflies were kept in liquid nitrogen immediately after treatment and were stored at −80 °C until use. Fifty whiteflies were transferred into a tissue grinding tube with 200 μL cell lysis buffer (20 Mm Tris, pH 7.5, 150 mM NaCl, 1% Triton X-100). After grinding, the lysate was centrifuged at 4 °C and the supernatant was used for enzymatic assays. The activity of the MnSOD was determined using the CuZn/Mn-SOD Assay Kit (Beyotime, Shanghai, China). During the assay, CuZnSOD inhibitor A and B were added in the sample according to the manufacturer’s protocol, so the result of the assay was only about MnSOD. The detailed procedures can be referred to in the protocol (Beyotime, Shanghai, China). Each treatment was replicated for three times. The statistical analysis is described in “Thermostability of purified Bt-mMnSOD”.

### 4.8. Effect of Host Shift on the MnSOD Activity of the Whitefly

Whiteflies collected from cotton plants were transferred to tobacco plants and new cotton plants (control). After 0 (control), 1, 3 and 5 days, 50 whiteflies were collected in a 5 mL tube from tobacco and new cotton plants, respectively. The whiteflies were then treated with liquid nitrogen and stored at −80 °C. Each treatment was replicated for three times. The method used for preparing samples and the detection of enzyme activity is described in “Effect of thermal stresses on the MnSOD activity of the whitefly”. The statistical analysis is described in “Thermostability of purified Bt-mMnSOD”.

### 4.9. Effect of Pesticide Treatment on MnSOD Activity of the Whitefly

Three cotton plants were sprayed with 15 mL of imidacloprid (Dashifeng, Beijing, China). The concentration of active ingredients was 20 mg/L. After 6 h, two thousand adult whiteflies were transferred to these cotton plants. Thereafter, 50 whiteflies were collected in a 5 mL tube after 0 (control), 6, 12 and 24 h, respectively. The whiteflies were then treated with liquid nitrogen and stored at −80 °C. Each treatment was replicated three times. The method used for preparing samples and the detection of enzyme activity is described in “Effect of thermal stresses on the MnSOD activity of the whitefly”. The statistical analysis is described in “Thermostability of purified Bt-mMnSOD”.

## 5. Conclusions

In this study, a mitochondrial manganese superoxide dismutase from the whitefly MEAM1 was cloned and characterized for the first time. Our results elucidated the important roles of MnSOD in the cellular stress responses and anti-oxidative processes of the whitefly MEAM1. The *in vivo* activity of Bt-mMnSOD under various conditions indicated the strong adaptive capacity of the invasive whitefly MEAM1 to extreme temperatures, host plants shift and insecticide treatment, which might contribute to its successful global invasion and continuous displacement of indigenous whitefly species. Future studies will be necessary to investigate functions of more antioxidant enzymes to gain a better understanding of the antioxidant mechanism in the whitefly species under various stresses.

## Figures and Tables

**Table 1:** Sequences of MnSOD used in this study.

Species	Protein	Abbreviation	GenBank no.
*Homo sapiens*	mitochondrial MnSOD	*H. sapiens*-mMnSOD	P04179.2
*Cavia porcellus*	mitochondrial MnSOD	*C. porcellus*-mMnSOD	XM_003466367.1
*Pongo abelii*	mitochondrial MnSOD	*P. abelii*-mMnSOD	NM_001133563.1
*Taeniopygia guttata*	mitochondrial MnSOD	*T. guttata*-mMnSOD	DQ214967.1
*Gallus gallus*	mitochondrial MnSOD	*G. gallus-*mMnSOD	NM_204211.1
*Melopsittacus undulatus*	mitochondrial MnSOD	*M. undulatus-*mMnSOD	AY241394.1
*Danio rerio*	mitochondrial MnSOD	*D. rerio-*mMnSOD	NP_956270.1
*Hemibarbus mylodon*	mitochondrial MnSOD	*H. mylodon-*mMnSOD	ACR23311.1
*Hypophthalmichthys nobilis*	mitochondrial MnSOD	*H. nobilis-*mMnSOD	ADM26563.1
*Bemisia tabaci*	mitochondrial MnSOD	*B. tabaci-*mMnSOD	JQ867105
*Acyrthosiphon pisum*	mitochondrial MnSOD	*A. pisum-*mMnSOD	NM_001246048.1
*Caenorhabditis elegans*	mitochondrial MnSOD	*C. elegans-*mMnSOD	D12984.1
*Ancylostoma duodenale*	mitochondrial MnSOD	*A. duodenale-*mMnSOD	FJ465146.1
*Haliotis discus discus*	mitochondrial MnSOD	*H. discus-*mMnSOD	DQ821491.1
*Mizuhopecten yessoensis*	mitochondrial MnSOD	*M. yessoensis-*mMnSOD	AB222783.1
*Callinectes sapidus*	mitochondrial MnSOD	*C. sapidus-*mMnSOD	AF264029.1
	cytosolic MnSOD	*C. sapidus*-cMnSOD	AAF74771.1
*Scylla paramamosain*	mitochondrial MnSOD	*S. paramamosain-*mMnSOD	FJ605170.2
	cytosolic MnSOD	*S. paramamosain*-cMnSOD	ADA63848.1
*Portunus trituberculatus*	cytosolic MnSOD	*P. trituberculatus*-cMnSOD	FJ031018.1
*Fenneropenaeus chinensis*	cytosolic MnSOD	*F. chinensis*-cMnSOD	ACS49842.1
*Litopenaeus vannamei*	cytosolic MnSOD	*L. vannamei*-cMnSOD	DQ005531.1
